# 14-3-3 proteins regulate Tctp–Rheb interaction for organ growth in *Drosophila*

**DOI:** 10.1038/ncomms11501

**Published:** 2016-05-06

**Authors:** Thao Phuong Le, Linh Thuong Vuong, Ah-Ram Kim, Ya-Chieh Hsu, Kwang-Wook Choi

**Affiliations:** 1 Department of Biological Sciences, Korea Advanced Institute of Science and Technology, 291 Daehak-ro, Yuseong-gu, Daejeon 305-701, Korea; 2 Department of Stem Cell and Regenerative Biology, Harvard University, Sherman Fairchild 358A, 7 Divinity Avenue Cambridge, Massachusetts 02138, USA; 3 Present address: Department of Developmental and Regenerative Biology and Graduate School of Biomedical Sciences, Icahn School of Medicine at Mount Sinai, New York, New York 10029, USA

## Abstract

14-3-3 family proteins regulate multiple signalling pathways. Understanding biological functions of 14-3-3 proteins has been limited by the functional redundancy of conserved isotypes. Here we provide evidence that 14-3-3 proteins regulate two interacting components of Tor signalling in *Drosophila*, translationally controlled tumour protein (Tctp) and Rheb GTPase. Single knockdown of 14-3-3ɛ or 14-3-3ζ isoform does not show obvious defects in organ development but causes synergistic genetic interaction with *Tctp* and *Rheb* to impair tissue growth. 14-3-3 proteins physically interact with Tctp and Rheb. Knockdown of both 14-3-3 isoforms abolishes the binding between Tctp and Rheb, disrupting organ development. Depletion of 14-3-3s also reduces the level of phosphorylated S6 kinase, phosphorylated Thor/4E-BP and cyclin E (CycE). Growth defects from knockdown of 14-3-3 and Tctp are suppressed by CycE overexpression. This study suggests a novel mechanism of Tor regulation mediated by 14-3-3 interaction with Tctp and Rheb.

## Abstract (web summary)

14-3-3 proteins regulate several signalling pathways but often act redundantly; however, the molecular mechanisms behind such redundancy are unclear. Here, the authors show that 14-3-3 proteins regulate two interacting components of Tor signalling in *Drosophila*, Tctp and Rheb, disrupting organ development.

## Graphical abstract

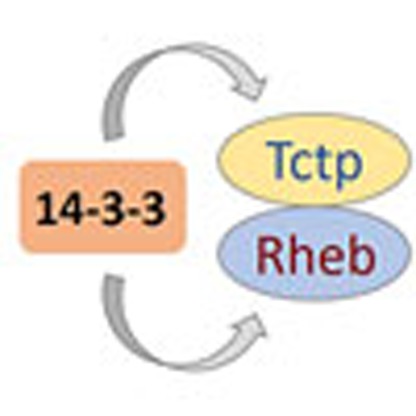

The Tor signalling pathway is important for the regulation of cell growth, proliferation, survival and metabolism in eukaryotes. Abnormal regulation of Tor signalling can cause various diseases including cancer and diabetes. Tor is the catalytic kinase subunit of the TORC1 complex that consists of several regulatory components such as Raptor and Pras40. The TORC1 complex integrates extracellular signalling and intracellular processes^1^. Growth factors like insulin activate Pi3k, leading to Akt activation. Akt is known to phosphorylate tuberous sclerosis complex 2 (Tsc2) to inhibit its GTPase-activating protein (GAP) activity for Rheb GTPase^2^,^3^. Hence, inactivation of the Tsc1/2 complex promotes Rheb function to activate Tor kinase^4^. A key role of Tor kinase is to stimulate protein synthesis for cell growth by phosphorylating ribosomal S6 kinase (S6k) and Thor (*Drosophila* 4E-BP). Lack of S6k results in a reduction of the cell size but not the cell number^5^. In *Drosophila, Rheb* and *Tor* kinase mutants show cell cycle arrest^6^,^7^. Loss of Tor in imaginal discs leads to a reduced cell proliferation and cyclin E (CycE), the S-phase regulator. Further, the cell cycle arrest in *Tor* mutants can be rescued by overexpressing CycE^7^, indicating that Tor signalling is required for the regulation of the CycE level, thus affecting cell proliferation.

Recent studies have shown that translationally controlled tumour protein (Tctp) is involved in Tor signalling^8^,^9^,^10^,^11^,^12^,^13^. Tctp is a family of evolutionarily conserved proteins involved in a number of fundamental processes, including cell proliferation, apoptosis and DNA damage control^14^,^15^,^16^,^17^. Tctp is upregulated in cancer cells, and its reduction results in the reversion of tumour phenotypes, implicating its role in tumorigenesis and tumour reversion^14^,^18^,^19^. In *Drosophila*, Tctp is required for organ growth by promoting Rheb function for Tor signalling as a guanine nucleotide exchange factor^8^. The role of Tctp in Tor signalling has also been reported in other organisms^9^,^10^,^11^,^12^,^13^. The function of Tctp family proteins in growth regulation seems to be conserved since *Drosophila* growth defects by Tctp knockdown can be restored by human Tctp^8^. Remarkably, functional conservation has also been found between animals and plants. For instance, *Drosophila* Tctp rescues embryonic lethality and cell proliferation defects in *Arabidopsis* Tctp (AtTctp) loss-of-function mutant. Consistent with this interspecies complementation, *Drosophila* Rheb not only interacts with Tctp but also with the plant AtTctp^13^. Since Rheb is essential for Tor activation, it is important to understand how the interaction between Tctp and Rheb is regulated.

Interestingly, 14-3-3 proteins interact with multiple regulators of Tor signalling such as Tsc2, Pras40 and Raptor in mammalian cells^20^,^21^,^22^,^23^,^24^. 14-3-3 proteins are conserved adaptor molecules that control diverse signalling pathways^25^,^26^, but their function *in vivo* has not been well characterized. Loss-of-function studies in *Drosophila* indicated that Pras40 is only required in ovary but not in other tissues^27^. Thus, it is important to identify physiologically critical functions of 14-3-3s and their interacting proteins in animal models. In *Drosophila*, 14-3-3 proteins are encoded by two genes, *14-3-3ɛ* and *14-3-3ζ* (also named *leo* for *leonardo*)^28^. Null mutations of either *14-3-3ɛ* or *14-3-3ζ* lead to embryonic lethality. However, some *14-3-3ɛ* homozygous mutants are viable, because lack of 14-3-3ɛ is compensated by elevated level of 14-3-3ζ protein^29^. *Drosophila 14-3-3* genes participate in Ras/Mapk signalling and neuronal differentiation^30^,^31^. They are also known to modulate FoxO-mediated apoptosis^32^, Hippo signalling^33^,^34^, and cell cycle regulation in syncytial nuclear division during embryogenesis^35^, but their roles in Tor signalling have not been studied.

In this study, we identify 14-3-3 proteins as binding partners for Tctp and Rheb. *14-3-3* genes show strong genetic interaction with *Tctp* and *Rheb*. 14-3-3 proteins physically interact with Tctp and Rheb. We provide evidence that 14-3-3 proteins are required for promoting the interaction between Tctp and Rheb. Loss of both 14-3-3 isoforms critically impairs organ development, and such defects are strongly suppressed by CycE. Our data suggest that 14-3-3 proteins regulate the interaction between Tctp and Rheb for organ growth, providing novel insights into their functions in Tor signalling.

## Results

### *Tctp* and *Rheb* interact genetically with *14-3-3* genes

**Figure 1:**
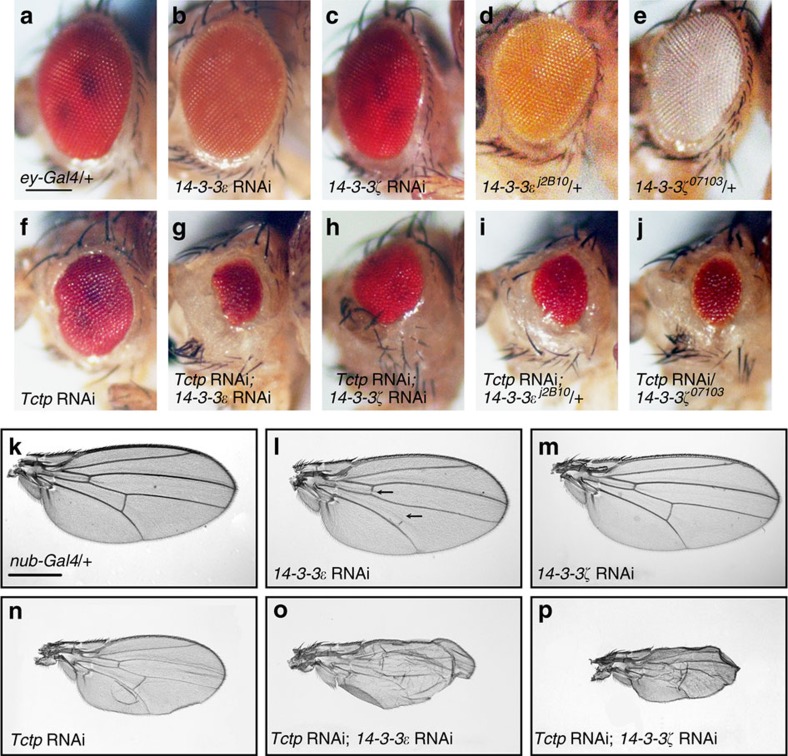
*Tctp* and *Rheb* interact genetically with *14-3-3*. (**a**–**j**) Genetic interaction between *Tctp* and *14-3-3* in eye. (**a**) Adult fly with one copy of *ey-Gal4* shows normal eye. (**b**,**c**) Knockdown of 14-3-3ɛ (**b**) or 14-3-3ζ (**c**) alone shows no obvious effect on eye. (**d**,**e**) Heterozygotes *14-3-3ɛ*^*j2B10*^/+ (**d**) or *14-3-3ζ*^*07103*^/+ (**e**) show normal eyes. (**f**) Knockdown of Tctp results in small and rough eye phenotype. (**g**,**h**) Knockdown of both Tctp and 14-3-3ɛ (**g**) or 14-3-3ζ (**h**) strongly enhances the eye phenotype caused by *Tctp* RNAi alone. (**i**,**j**) One copy of *14-3-3ɛ*^*j2B10*^ (**i**) or *14-3-3ζ*^*07103*^ (**j**) enhances the *Tctp* RNAi phenotype. (**k**–**p**) Genetic interaction between *Tctp* and *14-3-3* in wing. (**k**) Control wing with one copy of *nub-Gal4* is normal. (**l**) Knockdown of 14-3-3ɛ affects the anterior and posterior crossveins, but has no effect on wing size. (**m**) Knockdown of 14-3-3ζ does not affect wing development. (**n**) *Tctp* RNAi results in mildly reduced and curled wing. (**o**,**p**) Double knockdown of Tctp and 14-3-3ɛ (**o**) or 14-3-3ζ (**p**) enhances *Tctp* RNAi phenotypes. RNAi knockdown was induced by *ey-Gal4* (**a**–**c**,**f**–**j**) and *nub-Gal4* (**k**–**p**). Scale bar, 200 μm (**a**–**j**); 400 μm (**k**–**p**).

We have attempted to identify new genes interacting with *Tctp* by searching for genetic modifiers of the reduced eye phenotype caused by *Tctp* RNA interference (RNAi) using Gal4/UAS system^36^. *UAS-Tctp* RNAi and *eyeless (ey)-Gal4* were used to induce Tctp silencing mainly in undifferentiated cells of eye imaginal disc^8^. Knockdown of either 14-3-3ɛ or 14-3-3ζ alone did not show gross abnormalities in the eye (Fig. 1b,c). In contrast, *14-3-3ɛ* RNAi strongly enhanced the effects of Tctp knockdown in the eye, resulting in greatly reduced eyes (Fig. 1g) compared with a mild reduction by *Tctp* RNAi alone (Fig. 1f). We tested whether *14-3-3ζ* also shows genetic interaction with *Tctp*. Similar to *14-3-3ɛ* RNAi, knockdown of 14-3-3ζ enhanced the eye phenotypes of *Tctp* RNAi (Fig. 1h), while *14-3-3ζ* RNAi alone did not show obvious defects in the eye (Fig. 1c). *14-3-3ɛ* or *14-3-3ζ* mutant clones generated in eye and wing discs also appeared to be similar in the pattern of Elav (neuron-specific nuclear marker) and Discs-large (Dlg; cell membrane marker), respectively, compared with the surrounding wild-type tissues (Supplementary Fig. 1). To further confirm the synergistic genetic interaction between *Tctp* and *14-3-3* genes, we tested whether *Tctp* RNAi eye phenotypes can be enhanced by *14-3-3* mutations, *14-3-3ɛ*^*j2B10*^ and *14-3-3ζ*^*07103*^. Although *14-3-3ɛ*^*j2B10*^*/+* and *14-3-3ζ*^*07103*^*/+* heterozygotes did not show any obvious eye defect (Fig. 1d,e), these heterozygous conditions led to considerable enhancement of the *Tctp* RNAi eye phenotype (Fig. 1i,j).

We then examined whether the genetic interaction between *Tctp* and *14-3-3* genes can also be seen in another organ. When *nubbin (nub)-Gal4* was used to drive *Tctp* RNAi in developing wing disc, it resulted in consistent reduction and mild wrinkling of adult wings (Fig. 1n). Knockdown of either 14-3-3ɛ or 14-3-3ζ did not reduce the wing size (Fig. 1l,m), although it showed partial loss of crossveins (Fig. 1l). However, *14-3-3* RNAi strongly enhanced the effects of Tctp knockdown, causing reduction and severe wrinkling of wings (Fig. 1o,p). We performed similar genetic tests with two additional independent *Tctp* RNAi lines and obtained similar results, as shown in Supplementary Fig. 2. These RNAi lines driven by *ey-Gal4* or *nub-Gal4* resulted in reduced eye and wing, respectively (Supplementary Fig. 2d,g,m,p). Both *14-3-3ɛ* RNAi and *14-3-3ζ* RNAi enhanced the eye/wing phenotypes caused by *Tctp* RNAi (Supplementary Fig. 2d–i,m–r). These data indicate that *14-3-3* and *Tctp* genetically interact in both eye and wing development.

**Figure 2:**
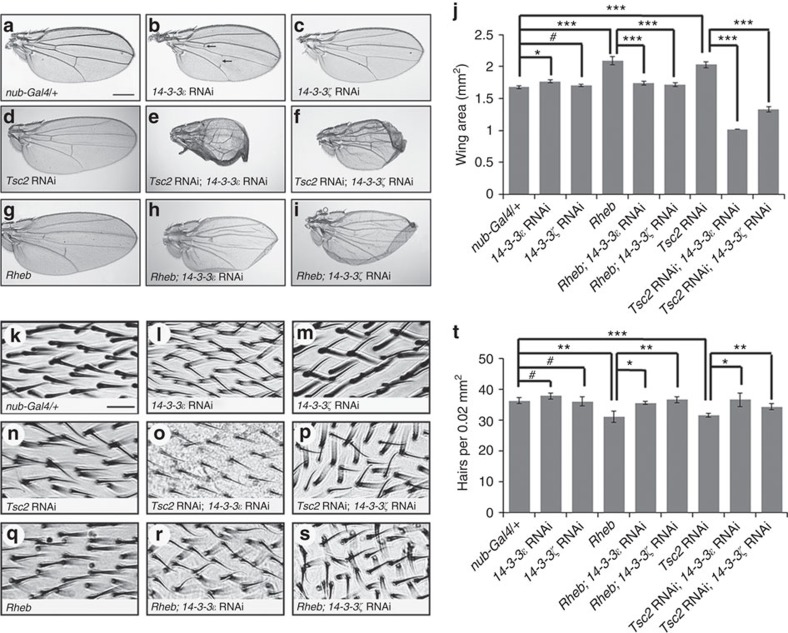
Genetic interaction of *14-3-3* with *Tsc2* and Rheb in the wing. (**a**–**f**) *14-3-3* shows genetic interaction with *Tsc2*. (**a**) Control wing with one copy of *nub-Gal4* is normal. (**b**,**c**) Knockdown of 14-3-3ɛ (**b**) or 14-3-3ζ (**c**) does not affect wing size. (**d**) *Tsc2* RNAi by *nub-Gal4* causes enlarged wing. (**e**,**f**) Reduction of 14-3-3 levels in *Tsc2* RNAi background results in inhibition of overgrowth and shape changes in wing. (**g**–**i**) Genetic interaction between *Rheb* and *14-3-3* in the wing. Overexpression of Rheb results in overgrowth of wing (**g**). (**h**,**i**) Knockdown of 14-3-3ɛ (**h**) or 14-3-3ζ (**i**) suppresses the effects of Rheb overexpression. RNAi or overexpression was induced by *nub-Gal4* (**a**–**i**). Scale bar, 400 μm (**a**–**i**). (**j**) Quantification of wing sizes shown in **a**–**i**. Knockdown of one isoform of 14-3-3 does not affect wing size compared with the control (*nub-Gal4/+*). Knockdown of Tsc2 or overexpression of Rheb increases the wing size about 20% compared with wild-type. Knockdown of one 14-3-3 isoform suppresses overgrowth phenotype caused by Rheb overexpression. Knockdown of one 14-3-3 isoform and Tsc2 results in about 30% reduction of wing size compared with the wild-type size. Error bars are s.d. *n*=10. *P<0.01. ***P<0.0001. ^#^*P*>0.05 (t-test). (**k**–**s**) High magnification images of flat areas of adult wings with indicated genotypes. Scale bar, 15 μm. (**k**) Wild-type wing; (**l**,**m**) Knockdown of 14-3-3ɛ (**l**) or 14-3-3ζ (**m**). (**n**) Knockdown of Tsc2; (**o**,**p**) Knockdown of both Tsc2 and 14-3-3ɛ (**o**) or 14-3-3ζ (**p**); (**q**) Overexpression of Rheb; (**r**,**s**) Overexpression of Rheb combined with knockdown of 14-3-3ɛ (**r**) or 14-3-3ζ (**s**). (**t**) Quantification of hair numbers within the same area of wings (0.02 mm^2^). Knockdown of one isoform of 14-3-3 does not affect the hair number. Knockdown of Tsc2 or overexpression of Rheb causes about 20% reduction in the wing hair number compared with wild-type. Knockdown of one isoform of 14-3-3 combined with *Tsc2* RNAi or Rheb overexpression results in similar wing hair numbers as wild-type. Error bars are s.d. *n*=10. *P<0.01. **P<0.001. ***P<0.0001. ^#^*P*>0.05 (t-test).

In Tor signalling, Tsc1/2 complex functions as GAP towards Rheb^37^,^38^. Thus, we tested whether *14-3-3* genes genetically interact with *Tsc2* in the wing model. Control single knockdown of either isoform of 14-3-3 in the wing using *nub-Gal4* did not affect the wing size (Fig. 2b,c), as also shown in Fig. 1. Knockdown of Tsc2 in the wing led to 19.5±0.05% tissue enlargements by overgrowth (Fig. 2d,j, *n*=10). Remarkably, silencing of either 14-3-3ɛ or 14-3-3ζ strongly inhibited the wing growth caused by *Tsc2* RNAi, even showing severe undergrowth of wings (Fig. 2e,f). Next, we examined whether *14-3-3* genes might also interact genetically with *Rheb*. Overexpression of Rheb by *nub-Gal4* induced 23±0.06% increase in wing size (Fig. 2g,j, *n*=10). Knockdown of 14-3-3ɛ inhibited the wing overgrowth caused by Rheb overexpression (Fig. 2h). Enlarged wing by Rheb overexpression was also suppressed by *14-3-3ζ* RNAi (Fig. 2i). This suppression by either one of *14-3-3* RNAi resulted in mild curling at the wing margin, but the wing size was consistently reduced to that of the wild-type control (Fig. 2j). We also tested the effects of *14-3-3* RNAi on cell size. Because each wing cell generates a single hair, the hair density within a given area provides an approximate estimation of cell size. Knockdown of either 14-3-3ɛ or 14-3-3ζ did not noticeably affect the wing hair density (Fig. 2k–m). The hair density was decreased by 13.1±0.58% in *nub>Tsc2* RNAi wings (Fig. 2n,t, *n*=10) and by 14.5±1.83% in *nub>Rheb* wings (Fig. 2q,t, *n*=10), suggesting an increase in cell size. The decreased wing hair density by Tsc2 silencing or Rheb overexpression was suppressed by knockdown of either 14-3-3ɛ (Fig. 2o,r, *n*=10) or 14-3-3ζ (Fig. 2p,s, *n*=10).

### Tctp and Rheb physically interact with 14-3-3 proteins

**Figure 3:**
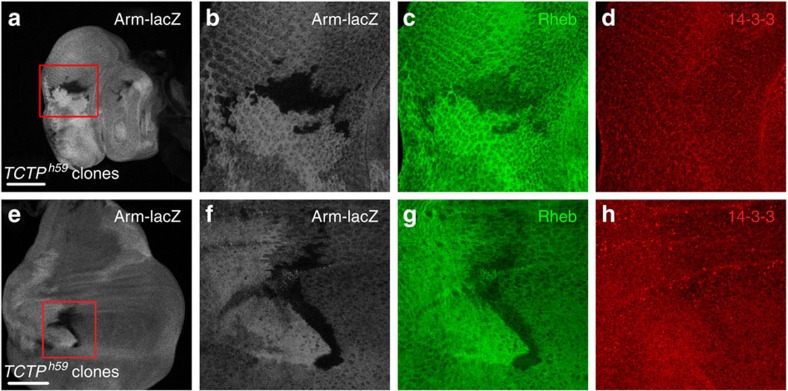
Effects of *Tctp* mutation on Rheb and 14-3-3s. (**a**–**d**) Clones of *Tctp*^*h59*^ null mutant cells in eye disc. (**e**–**h**) Clones of *Tctp*^*h59*^ null mutant cells in wing disc. These clones are marked by the absence of Arm-lacZ. In both organs, *Tctp* mutant clones show reduction of Rheb (**c**,**g**) but not 14-3-3s (**d**,**h**). Red rectangles in **a** and **e** indicate the enlarged regions shown in **b**–**d** and **f**–**h** respectively. Scale bar, 100 μm (**a**–**h**).

Strong genetic interaction of 14-3-3s with Tctp and Rheb suggests that they may function together to regulate organ development. Hence, we checked the expression of Tctp and Rheb in wing and eye discs from wild-type third instar larvae. For cellular localization of Rheb, we generated an anti-Rheb antibody which specifically detected Rheb protein in tissues (Supplementary Fig. 3a–f). Consistent with the physical interaction between Tctp and Rheb^8^, both proteins showed an overlapping expression pattern in eye disc (Supplementary Fig. 3g–i). Interestingly, loss of Tctp in mutant clones resulted in a reduction of the Rheb level in both eye and wing discs (Fig. 3c,g). We used an anti-14-3-3 antibody that specifically recognizes 14-3-3s but does not distinguish 14-3-3ɛ and 14-3-3ζ on western blot^32^. Immunostaining of wing discs with this antibody showed ubiquitous expression of 14-3-3s, but its level was reduced by *14-3-3ɛ* RNAi (Supplementary Fig. 3j–l), indicating the specificity of this antibody. Unlike Rheb, loss of Tctp did not cause obvious changes in the level of 14-3-3s (Fig. 3d,h).

**Figure 4:**
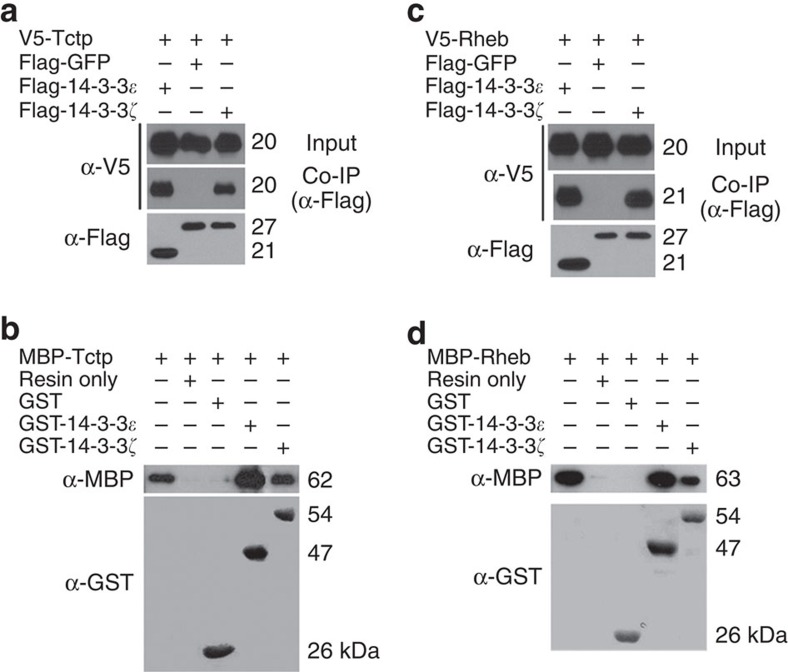
Tctp and Rheb physically interact with 14-3-3s. (**a**) Co-immunoprecipitation of 14-3-3s and Tctp. S2 cells were transfected with indicated genes. First western blot (WB) shows 5% input of V5-Tctp. Second blot shows V5-Tctp co-immunoprecipitated by Flag-14-3-3ɛ and Flag-14-3-3ζ but not by Flag-GFP. Third blot shows Flag-14-3-3ɛ, Flag-GFP and Flag-14-3-3ζ immunoprecipitated with anti-Flag. (**b**) Direct binding between 14-3-3s and Tctp. GST-14-3-3ɛ or GST-14-3-3ζ was used to pull-down MBP-Tctp as indicated. First blot shows MBP-Tctp proteins stained by anti-MBP. First lane indicates 5% input of MBP-Tctp used for pulldown. MBP-Tctp is pulled down by GST-14-3-3ɛ and GST-14-3-3ζ but not by GST. Second blot shows GST and GST-fusion proteins stained by anti-GST. (**c**) Co-immunoprecipitation of 14-3-3s and Rheb. First blot shows 5% input of V5-Rheb. Second blot shows V5-Rheb co-immunoprecipitated by Flag-14-3-3ɛ, and Flag-14-3-3ζ but not by Flag-GFP. Third blot shows Flag-14-3-3ɛ, Flag-GFP, and Flag-14-3-3ζ immunoprecipitated with anti-Flag. (**d**) Direct binding between 14-3-3s and Rheb. GST-14-3-3ɛ or GST-14-3-3ζ was used to pull-down MBP–Rheb as indicated. First blot shows MBP–Rheb stained by anti-MBP. First lane indicates 5% input of MBP–Rheb used for pulldown. MBP–Rheb is pulled down by GST-14-3-3ɛ and GST-14-3-3ζ but not by GST. Second blot shows GST and GST-fusion proteins stained by anti-GST.

Strong genetic interaction between Tctp and 14-3-3s, raised a possibility that they might physically interact. Co-immunoprecipitation (Co-IP) tests indicated that 14-3-3ɛ and 14-3-3ζ are associated with Tctp in a protein complex in S2 cell extracts (Fig. 4a). GST pull-down assays using bacterially expressed fusion proteins indicate that both 14-3-3ɛ and 14-3-3ζ bind to Tctp protein (Fig. 4b). These data suggest that 14-3-3ɛ and 14-3-3ζ directly interact with Tctp in a protein complex. As shown in Fig. 1, *14-3-3* genes show genetic interaction with *Rheb* as well as *Tctp*. Because Tctp and Rheb are directly associated, it is plausible that Rheb might also form a complex with 14-3-3. Co-IP assays using S2 cell extracts showed that Rheb co-immunoprecipitates with 14-3-3ɛ and 14-3-3ζ (Fig. 4c). Remarkably, Rheb was also pulled down by GST-14-3-3ɛ and GST-14-3-3ζ, indicating their direct binding *in vitro* (Fig. 4d).

### 14-3-3s are required for Tctp–Rheb interaction and growth

**Figure 5:**
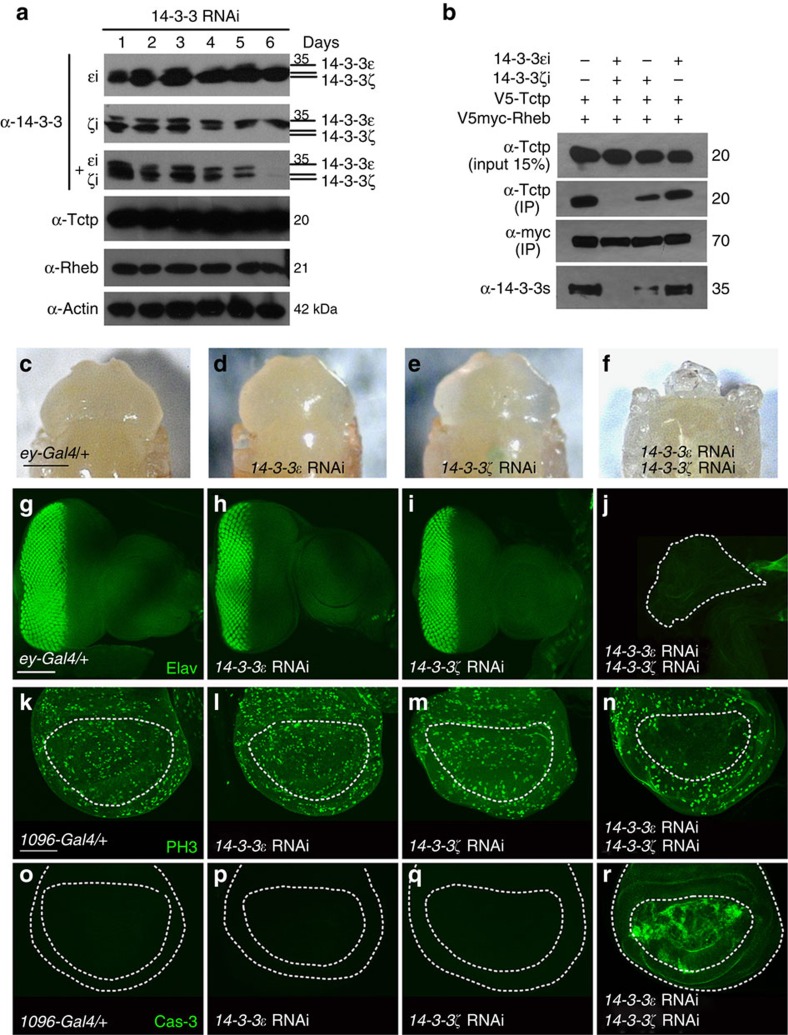
14-3-3s are required for the interaction between Tctp and Rheb and organ development. (**a**) Knockdown of 14-3-3s in S2 cells shows depletion of 14-3-3s levels after 6 days of RNAi treatment. ɛi, ζi and ɛi+ζi indicate RNAi for 14-3-3ɛ, 14-3-3ζ and both isoforms, respectively. Levels of Tctp and Rheb were not noticeably affected by 14-3-3 depletion. The treatment performed to detect Tctp, Rheb, and actin level was double knockdown of both isoforms of 14-3-3. (**b**) 14-3-3 depletion affects the interaction between V5-Tctp and V5myc-Rheb after 6 days of RNAi treatment. Anti-Myc antibody was used for IP. Tctp was detected by anti-Tctp antibody. Depletion of both isoforms abolished the Tctp–Rheb interaction. (**c**–**j**) Double knockdown of 14-3-3s causes loss of targeted tissues. *ey-Gal4* control (**c**) *14-3-3ɛ* RNAi (**d**) and *14-3-3ζ* RNAi (**e**) show normal pattern of eye-head in pupae. Knockdown of both 14-3-3 isoforms causes pupal lethality with loss of eye and head (**f**). (**g**–**j**) Pattern of Elav staining in larval eye discs from indicated genotypes. Knockdown of 14-3-3ɛ or 14-3-3ζ has no obvious effect on eye disc development (**h**,**i**). Knockdown of both isoforms results in small disc (the area marked with dotted line) with no retinal differentiation (**j**). (**k**–**n**) Double knockdown of 14-3-3s results in reduced proliferation. *MS1096-Gal4* control (**k**) *14-3-3ɛ* RNAi (**l**) and *14-3-3ζ* RNAi (**m**) show normal level of PH3 staining in wing disc. Knockdown of both 14-3-3 isoforms causes strong reduction of PH3 in the wing pouch (circled area) (**n**). (**o**–**r**) Caspase activation marked by anti-Caspase 3 (Cas-3) staining is not detected in *MS1096-Gal4* control (**o**) *14-3-3ɛ* RNAi (**p**) and *14-3-3ζ* RNAi (**q**). Double knockdown of both 14-3-3 isoforms results in strong activation of Cas-3 (**r**). Scale bar, 300 μm (**c**–**f**); 100 μm (**g**–**j**); 100 μm (**k**–**r**).

The data that 14-3-3s bind to both Tctp and Rheb led to a question if 14-3-3s are required for the interaction between Tctp and Rheb to form a complex. To test this hypothesis, we knocked down 14-3-3s in S2 cell culture, and checked whether reduced 14-3-3s might affect the interaction between Tctp and Rheb. 14-3-3 proteins were depleted in day 6th after treating S2 cell with *14-3-3s* RNAi (Fig. 5a). As shown in a previous study^29^, knockdown of one isoform of 14-3-3s resulted in upregulation of the other isoform to compensate the loss of the targeted isoform (Fig. 5a). When 14-3-3ɛ or 14-3-3ζ was depleted, there was no consistent change in the interaction between Tctp and Rheb, as assayed by co-IP. However, double knockdown of both 14-3-3 isoforms resulted in near complete loss of the binding between Tctp and Rheb (Fig. 5b). Levels of Tctp and Rheb were not altered by depleting 14-3-3s, indicating that the loss of Tctp–Rheb binding was not due to reduction of these proteins (Fig. 5a).

As shown above, single knockdown or loss-of-function mutation of either one of *14-3-3* genes did not affect organ growth. Since depletion of both 14-3-3 forms results in the disruption of physical interaction between Tctp and Rheb (Fig. 5b), we examined the effects of depleting both 14-3-3 isoforms in developing organs. In contrast to single knockdown that did not noticeably affect development (Fig. 5c–e), double knockdown of these two *14-3-3* genes resulted in pupal lethality. Removal of the pupal case from developing pupae revealed that double knockdown of both *14-3-3* genes causes loss of nearly entire eye and head tissues (Fig. 5f). Furthermore, dissection of third instar larvae showed massive disruption of eye–antenna discs, consistent with the loss of eye and head in pupal tissues (Fig. 5j). In contrast, single knockdown of either 14-3-3 isoform did not cause obvious defects in eye growth (Fig. 5h,i). Single RNAi knockdown in wing disc also did not affect cell proliferation or cell death marked by staining for phospho-Histone 3 (PH3; Fig. 5k–m) or activated Caspase 3 (Cas-3) (Fig. 5o–q), respectively. However, knockdown of both 14-3-3 isoforms strongly reduced PH3 staining (Fig. 5n) while increasing activated Caspase 3 (Fig. 5r). These results indicate that loss of both 14-3-3 isoforms critically impairs organ development by affecting cell proliferation and cell survival.

### Silencing 14-3-3s affects downstream targets of Tor

**Figure 6:**
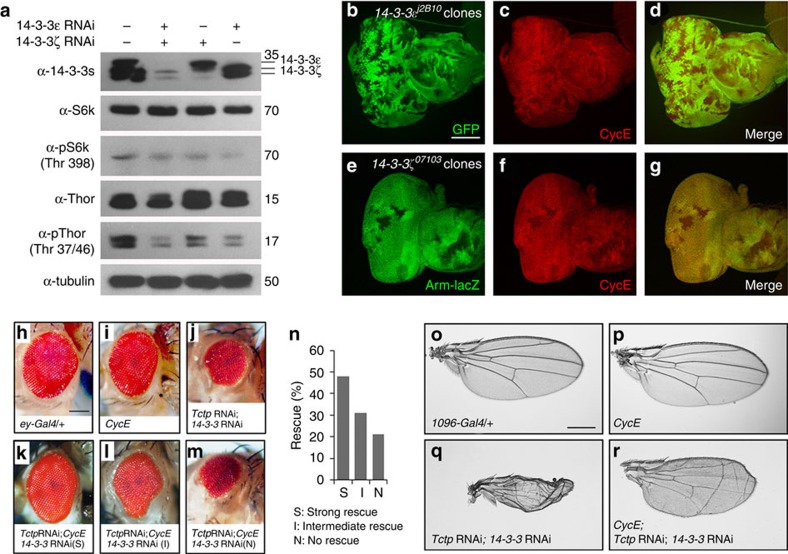
Effects of 14-3-3 RNAi on Tor targets and the suppression by CycE. (**a**) Effects of *14-3-3* RNAi on S6k and Thor. Either one of 14-3-3 isoforms or both 14-3-3s were knocked down in S2 cells. Single or double knockdown of 14-3-3s reduced the level of pS6k (Thr 398) but does not affect the level of S6k protein. Knockdown of both 14-3-3s, but not one isoform, causes slight reduction of Thor. In contrast, pThor (Thr 37/46) is strongly reduced by single or double knockdown of 14-3-3s. (**b**–**d**) Loss of 14-3-3s results in reduction of CycE level. (**b**) *14-3-3ɛ* mutant clones induced by *ey-flp* are marked by the absence of GFP. Mutant clones show strong reduction of CycE (**c**). (**e**–**g**) *14-3-3ζ* mutant clones induced by *hs-flp* are marked by the absence of Arm-lacZ. CycE is reduced in mutant clones (**f**). (**h**–**m**) Effects of CycE in eye. Control with one copy of *ey-Gal4* (**h**) or overexpression of CycE (**i**) shows normal eye. (**j**) Double knockdown of Tctp and 14-3-3 results in small and rough eye phenotype (<40% of wild-type eye size). (**k**–**m**) Overexpression of CycE rescues the small-eye phenotype resulting from double knockdown of Tctp and 14-3-3. The rescue is varied, ranging from strong (S, 81–100% of wild-type size) (**k**), intermediate (I, 41–80%) (**l**) to no rescue (N, 40% or lower) (**m**). (**n**) Quantification of rescue shown in **k**–**m**. More than 60 flies with the genotype of *ey>Tctp* RNAi/*CycE GFP*; *14-3-3* RNAi/+ were scored. (**o**–**r**) CycE suppresses the wing phenotypes of double knockdown of Tctp and 14-3-3. Control with one copy of *MS1096-Gal4* (**o**) or CycE overexpression (**p**) does not affect wing development. (**q**) Knockdown of both Tctp and 14-3-3 results in small and wrinkled wing. (**r**) Overexpression of CycE partially rescues the double-knockdown phenotype in 100% of tested flies. Scale bar, 100 μm (**b**–**g**); 200 μm (**h**–**m**); 400 μm (**o**–**r**).

Rheb activates Tor kinase, which leads to cell growth and proliferation through S6 kinase (S6k), Thor and CycE, respectively^7^,^39^. It has been shown that S6k phosphorylation is greatly reduced by Tctp silencing^8^. On the basis of genetic and physical interaction of 14-3-3s with Tctp and Rheb, we tested whether 14-3-3 knockdown can affect Tor activity by checking the levels of phosphorylated S6k and Thor in S2 cells. Either single or double knockdown of 14-3-3s resulted in similar reduction of pS6k detected by anti-pS6k (Thr 398) antibody, while there was little change in the total level of S6k protein. Phosphorylated Thor was strongly reduced by either single or double knockdown of 14-3-3s (Fig. 6a). Double knockdown of 14-3-3s resulted in some reduction of Thor protein levels while single knockdown did not.

To check the effects of *14-3-3* mutations on CycE, we generated *14-3-3ɛ* mutant clones in eye discs. These clones showed reduced levels of CycE (Fig. 6b–d). Similarly, *14-3-3ζ* mutant clones also resulted in a reduction of CycE level (Fig. 6e–g). However, such partial reduction of CycE by loss of one *14-3-3* isoform had little effect on the growth of mutant clones in the eye disc (Supplementary Fig. 1a–c,g–i), although reduced 14-3-3 causes abnormal differentiation of photoreceptor cells^30^,^31^.

### Defects by Tctp and 14-3-3 knockdown are suppressed by CycE

Because both Tctp and 14-3-3s affect Tor signalling, we tested whether double knockdown of Tctp and 14-3-3 can be suppressed by S6k and CycE. Growth defects from double knockdown of Tctp and 14-3-3 could not be suppressed by overexpressing S6k alone (Supplementary Fig. 4). Next, we tested the effects of overexpressing CycE. Single knockdown of either form of 14-3-3 strongly enhanced the effects of *Tctp* RNAi, as shown earlier (Fig. 1; Supplementary Fig. 2). Overexpression of CycE restored the small eye phenotype caused by knockdown of Tctp and one of 14-3-3s in ∼80% of flies examined (Fig. 6k–n), while CycE overexpression in the wild-type background did not increase the eye size (Fig. 6i). The small-wing phenotype resulting from double knockdown of Tctp and one of 14-3-3 isoforms was also partially rescued by CycE in nearly 100% flies examined (Fig. 6o–r).

We also examined whether CycE can suppress the effects of depleting both forms of 14-3-3 on wing development. Double knockdown of 14-3-3 isoforms in the wing pouch by *MS1096-Gal4* led to more than 90% pupal lethality and severe reduction of wing tissues in the surviving adult flies (Supplementary Fig. 5c). CycE overexpression in the wild-type background did not affect the wing size or survival (Supplementary Fig. 5b). In contrast, CycE overexpression in the 14-3-3 double knockdown condition suppressed the wing growth defect in >80% of the flies examined (Supplementary Fig. 5d–g), as well as pupal lethality (90% survival). These results suggest that reduced 14-3-3s synergize with partial loss of Tctp to affect the Tctp–Rheb interaction and Tor signalling, leading to a reduction of the CycE level.

## Discussion

Rheb plays a key role in Tor signalling by activating Tor kinase. Thus, how the Rheb function is regulated is an important issue. In this study, we have shown that 14-3-3 proteins play important roles in organ growth by affecting Tctp and Rheb for positive regulation of Tor signalling. Functional analysis of *14-3-3* genes *in vivo* has been limited due to their redundant roles. A clue for the role of 14-3-3s in Tor signalling *in vivo* was obtained from our finding of strong genetic interaction between Tctp and 14-3-3. Our data indicate that double knockdown of 14-3-3ɛ and 14-3-3ζ isoforms causes severe defects in organ growth, whereas silencing of either one of these isoforms has little effect. Interestingly, knockdown of 14-3-3ɛ or 14-3-3ζ together with *Tctp* RNAi results in synergistic growth defects. *14-3-3* genes also show strong genetic interaction with *Rheb* and *Tsc2* in which knockdown of one 14-3-3 isoform suppresses the wing overgrowth caused by Rheb overexpression or Tsc2 silencing. Knockdown of either 14-3-3ɛ or 14-3-3ζ has little effect on wing development. But together with Rheb overexpression, it results in smaller and curled-down wings in comparison with Rheb-overexpressed wing. Similarly, *14-3-3* RNAi did not simply suppress the overgrown wing phenotype of *Tsc2* RNAi to normal wing size. Instead, wings with knockdown of both 14-3-3 and Tsc2 are much smaller than the normal size of *14-3-3* RNAi wings. Currently, it is unknown how *14-3-3* RNAi negatively affects wing tissues under Rheb overexpression or Tsc2 knockdown conditions while *14-3-3* RNAi alone does not affect wing growth in the wild-type condition. It has been reported that tissues from *Tsc1* or *Tsc2* mutant patients show abnormal expression in a number of genes^40^. Therefore, it is possible that wing tissues with Rheb overexpression or *Tsc2* RNAi may have alterations in many intracellular events. Under these conditions, such tissues might become more sensitive to a reduction of 14-3-3 than wild-type tissues, resulting in abnormal or reduced wings. Alternatively, activated Tor signalling might affect other linked signalling pathways to induce cell death under certain conditions such as reduced 14-3-3. Some oncogenic genetic changes are known to promote apoptosis^41^. In case of Tor signalling, Tsc-deficient cells can constitutively activate Rheb-Tor signalling, which inhibits Akt signalling to promote cell death^42^. Therefore, it is possible that Tsc2 depletion or Rheb overexpression might lead to downregulation of other 14-3-3-dependent growth signalling pathways, thus resulting in wing growth defects when 14-3-3 levels are compromised. Identification of the mechanism underlying the synergistic interaction between 14-3-3 and Rheb/Tsc2 needs further studies.

In addition to genetic interaction, 14-3-3, Tctp, and Rheb physically interact with each other. Double knockdown of both 14-3-3 isoforms results in reduction of Tor downstream components such as phosphorylated S6k, phosphorylated Thor and CycE (Fig. 6a,b). In S2 culture cells, *14-3-3* RNAi did not affect the level of total S6k but it reduced the level of pS6k even by single knockdown of 14-3-3ɛ or 14-3-3ζ. Phosphorylated Thor was strongly reduced by knockdown of a single 14-3-3 isoform (Fig. 6a). On the contrary, single knockdown of 14-3-3ɛ did not strongly affect the interaction between Tctp and Rheb (Fig. 5b). These data seem to suggest that 14-3-3s may not affect TORC1 activity by modulating Tctp–Rheb binding. However, it is worth noting that 14-3-3s may also affect other Tor components such as Tsc2. Different 14-3-3 isoforms are known to inhibit Tsc2 in mammalian cells by binding to Tsc2 phosphorylated by Akt^43^. Therefore, although single knockdown of 14-3-3 may not strongly affect Tctp–Rheb interaction, depletion of a 14-3-3 isoform could elevate Tsc2 function to inhibit Rheb activity, thus reducing pS6k and pThor as shown in Fig. 6a. Our data for genetic interactions between *14-3-3* and *Tctp* (or *Rheb*) support the role of 14-3-3 affecting Tor signalling by interacting with Tctp and Rheb, although we do not exclude the possibility that 14-3-3s may also affect Tor signalling independent of Tctp–Rheb interaction.

An intriguing question is how single knockdown of 14-3-3 affects Tor signalling in S2 cells but not in animal tissues like eye/wing discs. Such difference might be due to cell- or tissue-specific function of Tor signalling^44^. For example, Tsc2 phosphorylation by Akt increases Tor signalling in mammalian cells. Although *Drosophila* Tsc2 is also phosphorylated by Akt in cultured S2 cells, Tsc2 phosphorylation does not play a critical role during normal development^45^. Thus, signalling events can vary between culture cells and intact organisms or even between different culture cell types. We noted that double knockdown of 14-3-3s fails to completely eliminate pS6k and pThor. Low levels of residual 14-3-3 (either 14-3-3ɛ or 14-3-3ζ) after RNAi treatment might be sufficient to keep some of TORC1 activity. It is also possible that Tor might be activated in part by Rheb-independent mechanisms in S2 cells as reported in mammalian cells^46^.

Mammalian Tctp proteins are known to be phosphorylated at several sites by Polo-like kinase 1 (ref. 47) and insulin signalling^48^. Mammalian Rheb is phosphorylated at Ser-130 by p38 regulated/activated protein kinase (Prak)^49^. Although *Drosophila* Tctp and Rheb have conserved Ser or Thr residues at the similar positions, no experimental evidence for phosphorylation has been reported. It seems that these serine or threonine sites do not belong to two conserved 14-3-3 binding motifs R[S/Ar]XpSXP and RX[Ar/S]XpSXP, where Ar is an aromatic amino acid. However, some 14-3-3 binding ligands have sequences diverged from these motifs or do not require phosphorylation for binding^50^. Therefore, it remains to be determined whether 14-3-3 binding to Tctp is mediated through phosphorylation.

Importantly, our data show that the binding between Tctp and Rheb is almost completely disrupted by knockdown of both 14-3-3 isoforms but not by a single knockdown. This suggests that 14-3-3 isoforms might play a redundant role to promote direct interaction between Tctp and Rheb. Since 14-3-3s bind directly to both Tctp and Rheb, Tctp–Rheb binding might be promoted, possibly by using the known dimerization of 14-3-3 proteins^29^,^50^. For such interactions to occur, these three proteins must be present in a stoichiometric ratio to form a proper complex. In our co-IP assays in S2 cells, the levels of endogenous 14-3-3 proteins were similar to the levels of exogenous Tctp and Rheb (Supplementary Fig. 6), suggesting that 14-3-3s, Tctp and Rheb are present in a stoichiometric ratio to form a direct complex. However, it is also possible that 14-3-3s might interact separately with Tctp and Rheb to promote the binding between Tctp and Rheb without forming a direct complex coupled by 14-3-3 proteins. Further studies are necessary to identify the precise mechanism of molecular interactions among these proteins.

Our study reveals a novel function of 14-3-3 for promoting the Tctp–Rheb interaction at the critical step of Tor activation. 14-3-3 proteins are also known to regulate Tor signalling by interacting with Tsc2, Pras40 and Raptor^20^,^21^,^22^,^23^,^24^, although *in vivo* functions of these interactions have not been studied in *Drosophila*. Thus, 14-3-3 isoforms may be involved in more steps than those known so far, both up and/or downstream of Tor kinase. It would be important to study how such diverse 14-3-3 interactions with multiple Tor components contribute to generate signalling outputs for cell growth and proliferation. Nonetheless, the new function of 14-3-3 isoforms for the interaction between Tctp and Rheb is crucial for organ growth in *Drosophila*. It would be interesting to see whether the function of 14-3-3s shown in this study is conserved in vertebrates and whether loss of a functionally redundant human 14-3-3 isoform can synergize with partial defects in Tor signalling components, leading to more severe pathological conditions.

## Methods

### Fly genetics

*14-3-3* RNAi fly lines were obtained from the Vienna Drosophila Resource Center (VDRC) and the National Institute of Genetics (NIG). *14-3-3ɛ* RNAi lines were 31196R-4 and 31196R-3 (NIG). *14-3-3ζ* RNAi lines were 48724, 48725, 104496 (VDRC) and 17870R-2 (NIG). *14-3-3ɛ* loss-of-function mutant strain^30^ (*y*^*d2*^ *w*^*1118*^ *ey-flp; GMR-lacZ; FRT82B 14-3-3ɛ*^*j2B10*^*/TM6 Tb*) was from the Drosophila Genomics Resource Center (DGRC)*. 14-3-3ζ*^*07103*^ P-insertion lethal mutant strain^31^ (*w*; *FRT42D 14-3-3ζ*^*07103*^*/CyO)* was from the Bloomington Drosophila Stock Center*. Tctp* RNAi and mutant line *FRT82B Tctp*^*h59*^*/TM6 Tb* were as described^8^. Two other *Tctp* RNAi lines from VDRC were v45531 and v45532. Flies were grown at room temperature, unless stated otherwise.

For double knockdown of 14-3-3s, *MS1096-Gal4* and *UAS-14-3-3ɛ* RNAi were recombined on the first chromosome. *MS1096>14-3-3ɛ* RNAi recombinant females were crossed with male *UAS-14-3-3ζ* RNAi.

For double knockdown of Tctp and 14-3-3, *ey>Tctp* RNAi*/CyO* recombinants were crossed with *14-3-3ɛ* RNAi or *UAS-14-3-3ζ* RNAi. For rescue of double knockdown of 14-3-3s by CycE, *MS1094>14-3-3ɛ* RNAi recombinant female flies were crossed with *UAS-CycE GFP* on the second chromosome. Progeny from this cross was mated with *UAS-14-3-3ζ* RNAi on the third chromosome. Larvae showing green fluorescent protein (GFP) expression with the genotype *MS1096>14-3-3ɛ* RNAi*/+; CycE GFP/+; 14-3-3ζ* RNAi*/+* were selected to check phenotypes.

For rescue of double knockdown of Tctp and 14-3-3 by CycE, *ey>Tctp* RNAi*/CyO; 14-3-3ɛ* RNAi*/TM6 Tb* or *ey>Tctp* RNAi*/CyO; 14-3-3ζ* RNAi*/TM6 Tb* were selected based on eye phenotype. These recombinant flies were crossed with *UAS-CycE GFP*, and the progeny with GFP expression and without *TM6 Tb* balancer was kept for further analysis.

### Generation of mosaic clones in eye and wing discs

Mutant clones were generated by FLP-FRT method^51^. Clones of *14-3-3ɛ* mutation in eye discs were made using *14-3-3ɛ*^*j2B10*^ mutant fly line (*y*^*d2*^ *w*^*1118*^ *ey-flp*; *GMR-lacZ; FRT82B 14-3-3ɛ*^*j2B10*^*/TM6 Tb*) from DGRC. *14-3-3ɛ* mutant flies were crossed with *FRT82B ubi-GFP/TM3 Sb* fly (Bloomington). Eye discs from third instar larval progeny were dissected and immunostained with antibodies indicated in each figure. Mutant clones were identified by the absence of GFP.

To generate *14-3-3ɛ* mutant clones in wing discs, we crossed *hs-flp*; *FRT82B ubi-GFP/TM3 Sb* with *y*^*d2*^ *w*^*1118*^ *ey-flp*; *GMR-lacZ; FRT82B 14-3-3ɛ*^*j2B10*^*/TM6 Tb.* First instar larvae from this cross were heat shocked at 37 °C for 1 h and incubated at 25 °C until third instar stage. Wing discs were dissected for immunostaining. Mutant clones were identified by the absence of GFP staining. To generate *14-3-3ζ* mutant clones, *w*; *FRT42D 14-3-3ζ*^*07103*^*/CyO* flies were crossed with *hs-flp*; *FRT42D arm-lacZ*. First instar larvae were heat shocked as described above, and eye and wing discs from third instar larvae were dissected for immunostaining. Mutant clones were identified by the absence of LacZ staining. *Tctp* mutant clones were generated by crossing *hs-flp*; *FRT82B arm-lacZ* with *w; FRT82B Tctp*^*h59*^*/TM6B Tb.* First instar larvae were heat shocked as described above, and imaginal discs from third instar larvae were dissected for immunostaining.

### Generation of anti-Rheb antibody

Five-hundred forty-nine-base pair full-length Rheb complementary DNA (cDNA) including stop codon was cloned to pMAL-c2 vector. MBP–Rheb fusion protein was purified from bacteria and used for generating rat anti-Rheb antibody from Abclon. Anti-Rheb antibody was used at 1:500 for western blot and 1:100 for immunocytochemistry as described below.

### Immunocytochemistry

Third instar eye and wing imaginal discs were dissected in 1 × PBS, fixed in PLP (2% paraformaldehyde, 0.01 M periodate, 0.075 M lysine and 0.035 M phosphate buffer) for 30 min, and incubated with primary antibodies at 4 °C overnight. After washing, imaginal discs were incubated with secondary antibodies for 2 h at room temperature. First, antibodies were used as following: rat anti-Rheb at 1:100, rabbit anti-Tctp^8^ at 1:200, rabbit anti-14-3-3 (Invitrogen 51-0700) at 1:200, mouse anti-β-gal (DSHB 40-1a) at 1:50, rat anti-Elav (DSHB 7E8A10) at 1:100, rabbit anti-PH3 (Milipore) at 1:200, rabbit anti-Cas-3 (Cell Signaling 9661) at 1:300, sheep anti-GFP (Ab-direct Serotec 4745–1051) at 1:100, rabbit anti-CycE (Santa Cruz) at 1:100 and rabbit anti-Dlg (from Dr Kyung-Ok Cho) at 1:500. Secondary antibodies conjugated with Cy3, Cy5 or FITC (Molecular Probes) were: anti-rabbit Cy3, anti-rat FITC, anti-mouse Cy5, and anti-sheep FITC. Mounting solution was Vectashield with 4′,6-diamidino-2-phenylindole (Vector Laboratories H-1200). Fluorescent images were acquired using Carl Zeiss confocal microscope.

### *In vitro* GST pull-down assays

*14-3-3ɛ* cDNA (LD27892) and *14-3-3ζ* cDNA (RH61958) were from DGRC. Five-hundred eighty eight base pair of 14-3-3ɛ coding sequence (including stop codon) was amplified by PCR reaction and cloned into PGEX-4T1 vector. For 14-3-3ζ, 747 bp coding sequence (including stop codon) was cloned into PGEX-4T1 vector. Tctp- and Rheb-coding sequences were cloned into pMAL-c2 vector. Fusion tagged proteins were expressed in *BL21 Escherichia coli*. For GST pull-down assay, 20 μg of each MBP and GST-tagged protein were added to 500 μl pull-down buffer (20 mM Tris pH 7.5, 150 mM NaCl, 0.5 mM EDTA, 10% glycerol, 0.1% Triton X-100, 1 mM dithiothreitol, 1 mM Na_3_VO_4_, 10 mM NaF and a protease inhibitor cocktail (Roche 11 873 580 001)), followed by incubation overnight at 4 °C with Glutathione Excellose resin (Bioprogen). After 3 times of washing with cold pull-down buffer, 5 × SDS loading buffer was added and samples were boiled at 94 °C for 5 min, then centrifuged at 12,000 r.p.m for 5 min and loaded. For immunostaining of western blots, rabbit anti-MBP antibody (Santa Cruz) and secondary anti-rabbit antibody (Jackson) were used at 1:5,000 and 1:10,000.

### Co-immunoprecipitation assays

Coding sequences of 14-3-3s were cloned into Flag-tagged pRmHa-3 vector (DGRC). Tctp- and Rheb-coding sequences were cloned into pAC-V5HisA vector (Invitrogen). For co-IP between Tctp and Rheb, Rheb-coding sequence was cloned into pAC-V5mycHisA vector (Invitrogen). Co-IP assays were carried out using a standard protocol^52^ with minor modifications. DNA concentration was adjusted to 1 μg μl^−1^ before transfection. S2 cells were transfected with Flag-tagged 14-3-3 and V5-tagged Tctp (or V5-tagged Rheb) using Effectene reagent (Qiagen). After 24 h of transfection, 0.1 M CuSO_4_ was added to final concentration 0.5 mM to induce protein expression using pMT vector. S2 cells were incubated for additional 48 h, and extracted using CHAPS-based lysis buffer (0.1% CHAPS, 20 mM HEPES, 1 mM EDTA, 1 mM PMSF and a protease inhibitor cocktail (Roche)). Other steps were carried out according to the described protocol^52^. In brief, 50 μl of G-sepharose beads was washed and incubated with 2 μl of anti-Flag antibody (Sigma 1,804) in 500 μl of CHAPS-based lysis buffer for 2 h at room temperature. S2 cell lysates were incubated with 20 μl of G-sepharose beads to avoid non-specific binding between resin and protein extracts. The S2 cell lysates were then incubated with anti-Flag-bound G-sepharose beads overnight at 4 °C. After three times of 5-min washing with CHAPS-based lysis buffer, 20 μl of lysis buffer was mixed with 5 μl of 5 × SDS loading buffer. The samples were boiled at 94 °C for 5 min and centrifuged at 12,000 r.p.m for 5 min. Twenty microlitre of each sample was loaded on SDS gel for western blotting. Mouse anti-V5 (1:5,000, Invitrogen 46-0705) was used to detect V5-Tctp or V5-Rheb. Co-IP between V5myc-Rheb and V5-Tctp was carried out using anti-Myc (Abcam Ab9106) as bait to immunoprecipitate V5myc-Rheb. Co-immunoprecipitated V5-Tctp was detected by anti-Tctp antibody. Immunoprecipitated V5myc-Rheb was detected with rabbit anti-Myc at 1:5,000.

### RNAi treatment of S2 cells

S2 cells were treated with dsRNA following the protocol by Rogers *et al*.^53^ with minor modifications. Primers used for 14-3-3ɛ RNAi were 5′- TAATACGACTCACTATAGGGAGAAATGGTGGAGGCCATGAAG -3′ and 5′- TAATACGACTCACTATAGGGAGATTCAATGCCAAGCCCAAAC -3′. Primers used for 14-3-3ζ RNAi were 5′- TAATACGACTCACTATAGGCGAAGCATCCGCTAGAAAACAGC -3′ and 5′- TAATACGACTCACTATAGGTCGTTCAGTGTGTCCAGCTCGG -3′. Primers were designed using SnapDragon-dsRNA design (http://www.flyrnai.org/snapdragon). PCR reactions were carried out to amplify ∼500 bp target sequence of *14-3-3*. Reverse transcription reaction was performed using MEGAscript T7 (Ambion) and the products were purified with MEGAClear (Ambion). S2 cells cultured in M3 media (Sigma) with 10% insect medium supplement (Sigma) were seeded on 12-well plate with the density of 1 × 10^6^ cells per ml. For 14-3-3 RNAi treatment, RNA for 14-3-3ɛ and 14-3-3ζ was added to media at 5 and 10 μg ml^−1^, respectively. After 3 days from the first treatment, RNAs were added the second time on fourth day. S2 cells were collected each day and lysed in 200 μl of cold CHAPS-based lysis buffer for 30 min at 4 °C with rotating. Cell extracts were centrifuged at 12,000 r.p.m for 5 min at 4 °C. Five micro-liter of 5 × SDS loading buffer was added to 20 μl of sample. Total amount of 25 μl sample was boiled at 94 °C for 5 min, and 20 μl sample was loaded for western blot analysis. The following antibodies were used at the indicated dilution: rabbit anti-14-3-3 (Invitrogen 51-0700) at 1:1,000, rabbit anti-Tctp^8^ at 1:2,500; rat anti-Rheb at 1:500, and mouse anti-β-actin (Abcam Ab8224) at 1:5,000. For detecting S6k and phospho-S6k, mouse anti-S6k (ref. 8) and rabbit anti-pS6k (Thr 398) (Cell signaling 9209S) were used at the same 1:1,000 dilution, respectively. For detecting Thor protein and phosphorylated Thor, rabbit anti-Thor antibody^54^ and rabbit anti-pThor (Thr 37/46) (Cell Signaling 2855S) were used at 1:5,000 and 1:1,000, respectively.

All uncropped western blots can be found in Supplementary Fig. 7.

## Additional information

**How to cite this article:** Le, T. P. *et al*. 14-3-3 proteins regulate Tctp–Rheb interaction for organ growth in *Drosophila*. *Nat. Commun.* 7:11501 doi: 10.1038/ncomms11501 (2016).

## Supplementary Material

Supplementary Information Supplementary Figures 1-7
