# Supplementary material for: 14-3-3 proteins regulate Tctp–Rheb interaction for organ growth in Drosophila
Source: Nat Commun. 2016 May 6;7:11501. doi: 10.1038/ncomms11501 (PMC4859069; doi:10.1038/ncomms11501)
Supplement: Supplementary Information — Supplementary Figures 1-7 [file ncomms11501-s1.pdf]

Supplementary Figure 1

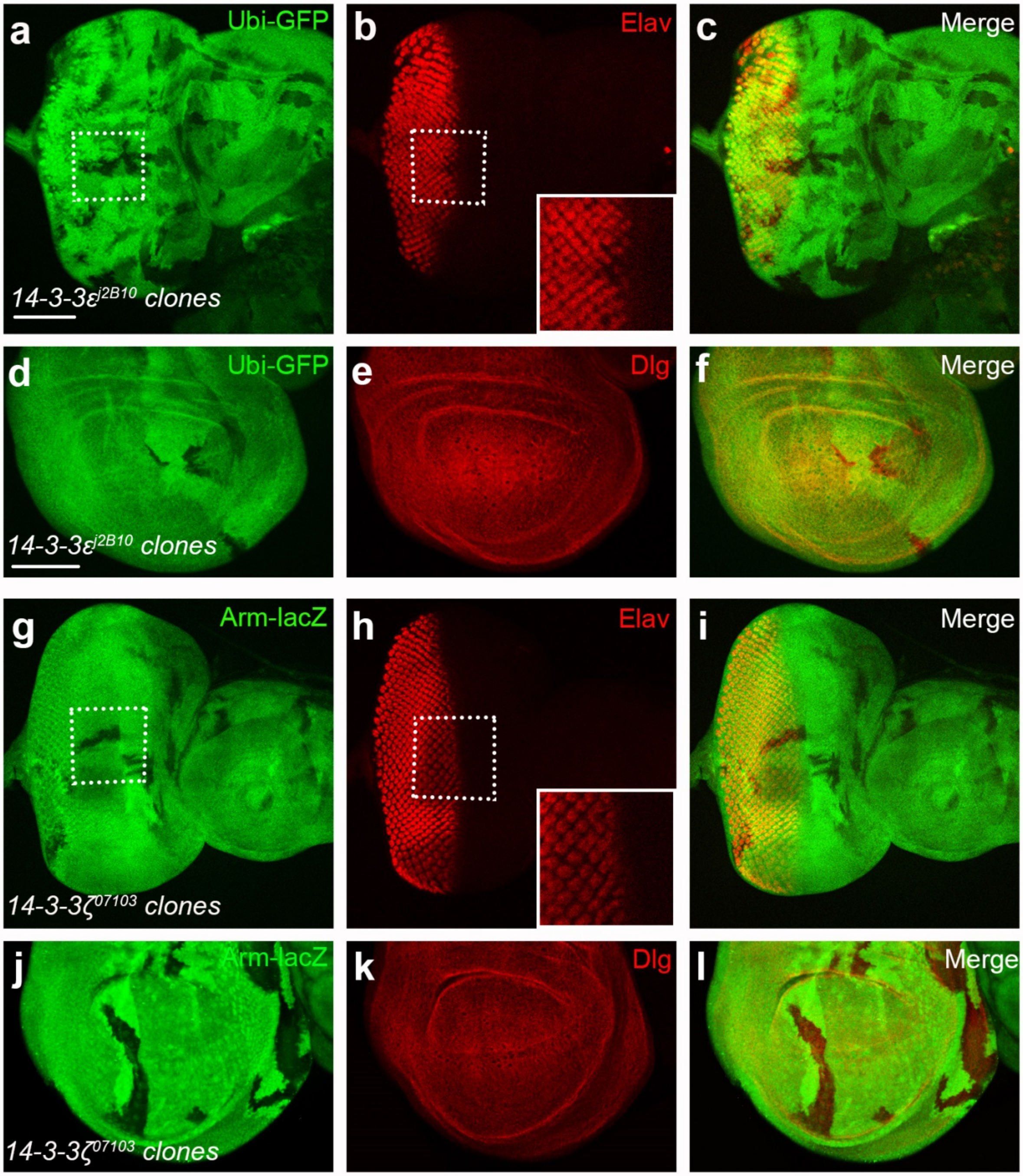

Supplementary Figure 1. Loss of function clones of *14-3-3ε* or *14-3-3ζ* show no significant effects on organ development. a-f, Eye and wing discs with clones of *14-3-3*ε^{j2B10} show no obvious defects in Elav and Dlg staining patterns, respectively. **g-l,** Eye and wing discs with clones of *14-3-3*ζ^{07103} develop normally with no obvious defects in Elav and Dlg patterns. Mutant clones were marked by loss of Ubi-GFP or Arm-lacZ, as indicated. Anti-Elav was used to mark differentiated neuronal cells in eye disc. Anti-Dlg was used to mark cell membrane in wing disc. Scale bar, 100 µm (**a-c, g-i**), 100 µm (**d-f, j-l**).

Supplementary Figure 2

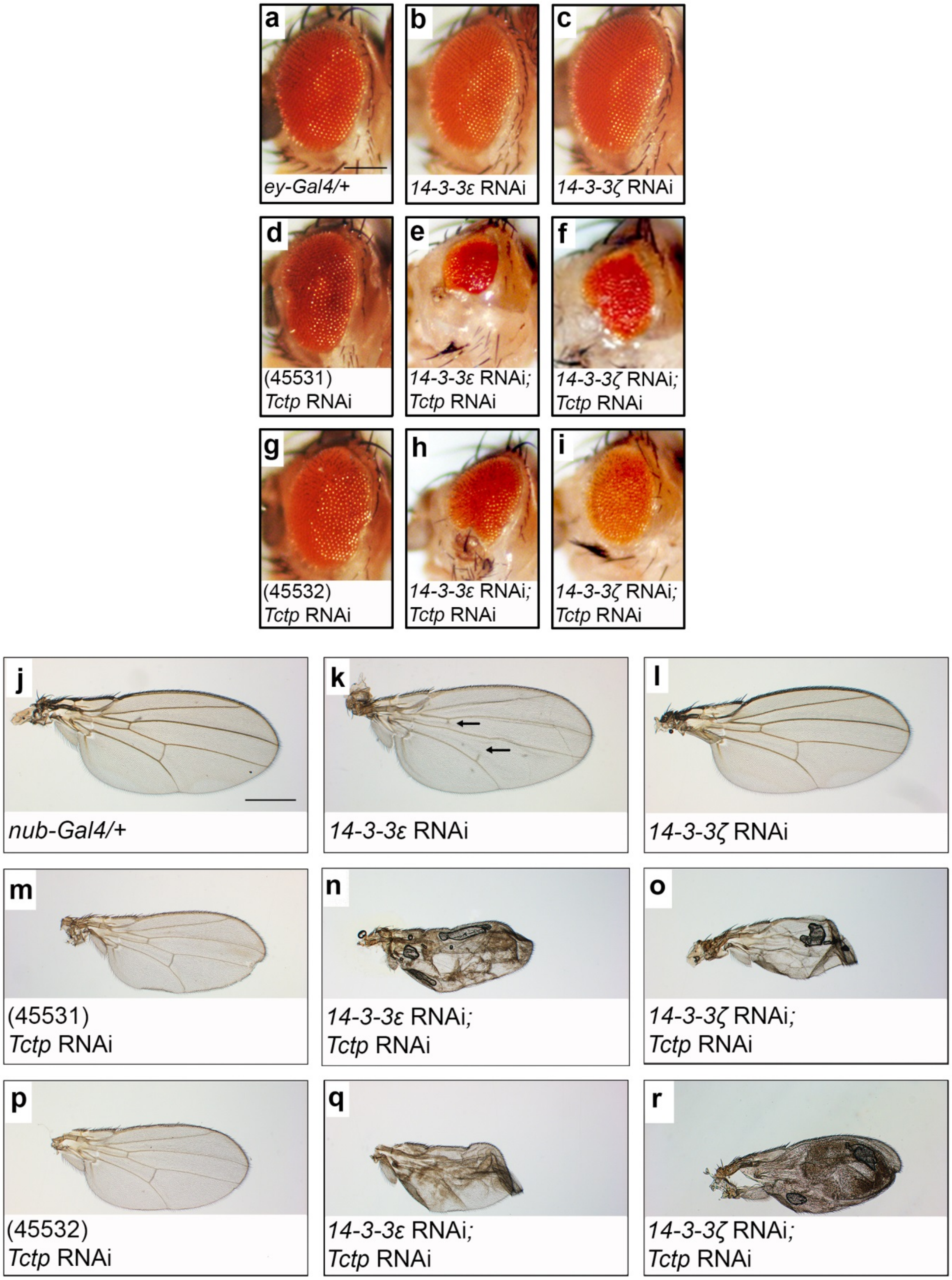

Supplementary Figure 2. Genetic interaction between *Tctp* with *14-3-3s* with two different *Tctp* RNAi lines. a-i, Genetic interaction between *Tctp* and *14-3-3* in adult eye. **a,** Adult fly with one copy of *ey-Gal4* shows normal eye. **b-c,** Knockdown of 14-3-3ε (**b**) or 14-3-3ζ (**c**) alone does not affect eye. **d,** Knockdown of Tctp using v45531 RNAi line (VDRC) results in small eye phenotype. **e-f,** Knockdown of both Tctp and 14-3-3ε (**e**) or 14-3-3ζ (**f**) strongly enhances the eye phenotype caused by *Tctp* RNAi alone. **g-i,** v45532 RNAi line (VDRC) gives similar results with v45531. **j-r,** Genetic interaction between *Tctp* and *14-3-3* in wing. **j,** Control wing with one copy of *nub-Gal4* is normal. **k,** Knockdown of 14-3-3ε affects the anterior and posterior cross veins (black arrows), but has no effect on wing size. **l,** Knockdown of 14-3-3ζ does not affect wing development. **m,** *Tctp* RNAi (v45531) results in mildly reduced and curled wing. **n-o,** Double knockdown of Tctp and 14-3-3ε (**n**) or 14-3-3ζ (**o**) enhances the *Tctp* RNAi phenotypes. **p-r,** Similar results are obtained with *Tctp* RNAi line v45532 (VDRC). Scale bar, 200 μm (**a-i**), 400 μm (**j-r**).

Supplementary Figure 3

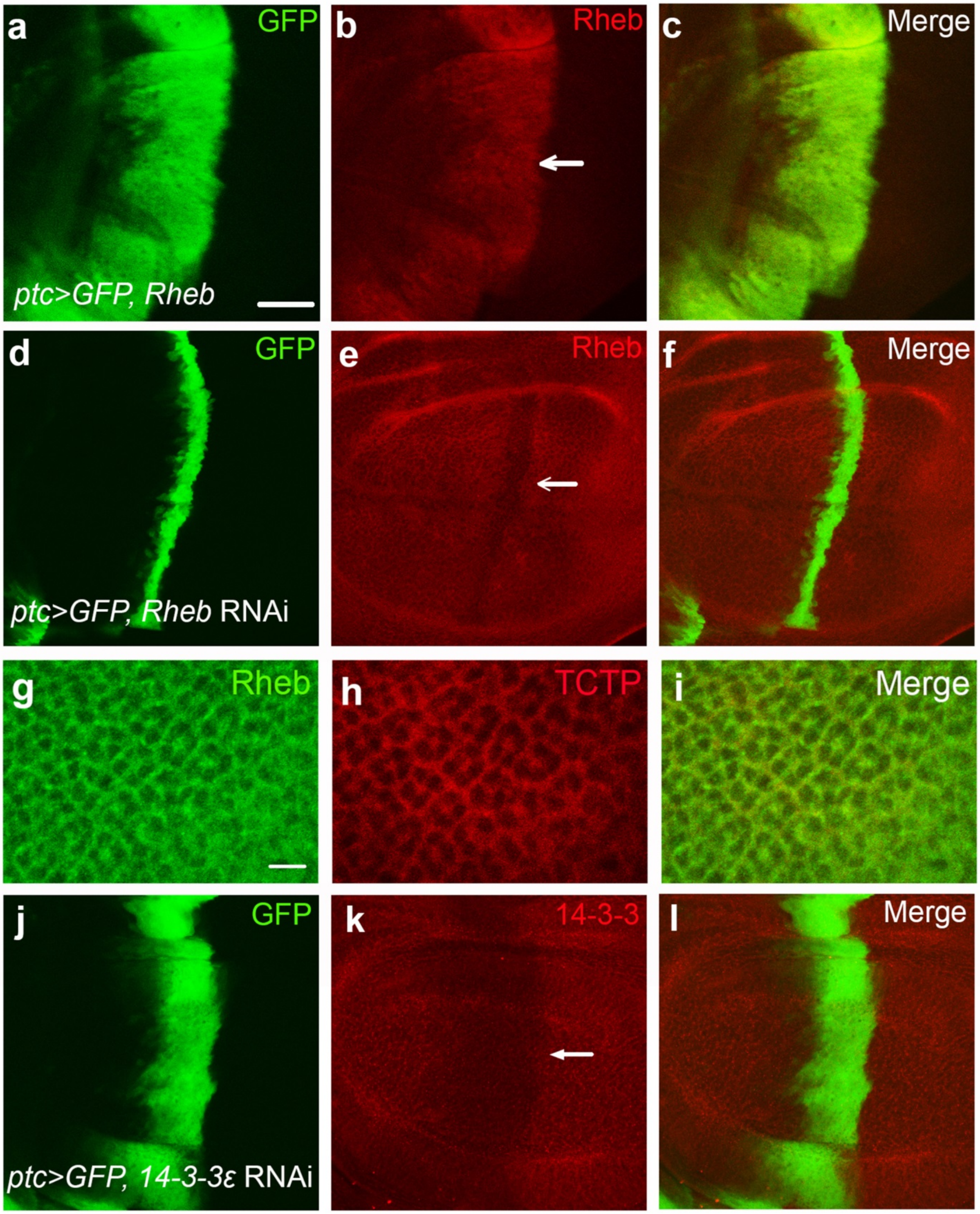

Supplementary Figure 3. Specificity of anti-Rheb and anti-14-3-3 antibodies. a-c, Rheb expression level was examined in wing disc. **a,** *patched (ptc)-Gal4 UAS-GFP* (*ptc>GFP)* was used to overexpress Rheb in the anterior-posterior boundary region of wing. **b,** Rheb is overexpressed in the *ptc* region (white arrow). The *ptc* domain is enlarged by Rheb overexpression. **c**, Merge of **a** and **b**. **d-f,** *ptc>GFP* was used to knockdown Rheb in the *ptc* region. **e,** Rheb staining is reduced in the *ptc* region. The width of the *ptc* domain is reduced by Rheb knockdown. **g-i**, Rheb and Tctp show an overlapping expression pattern in eye imaginal disc. Both are enriched in the boundaries between ommatidia and in the center of each ommatidium where photoreceptor cells contact together. **j-l,** 14-3-3 antibody staining is reduced in the *ptc* domain (GFP-positive region) by *14-3-3ε* RNAi (**k**), suggesting that the antibody is specific. Scale bar, 50 µm (**a-f, j-l**), 5 µm (**g-i**).

Supplementary Figure 4

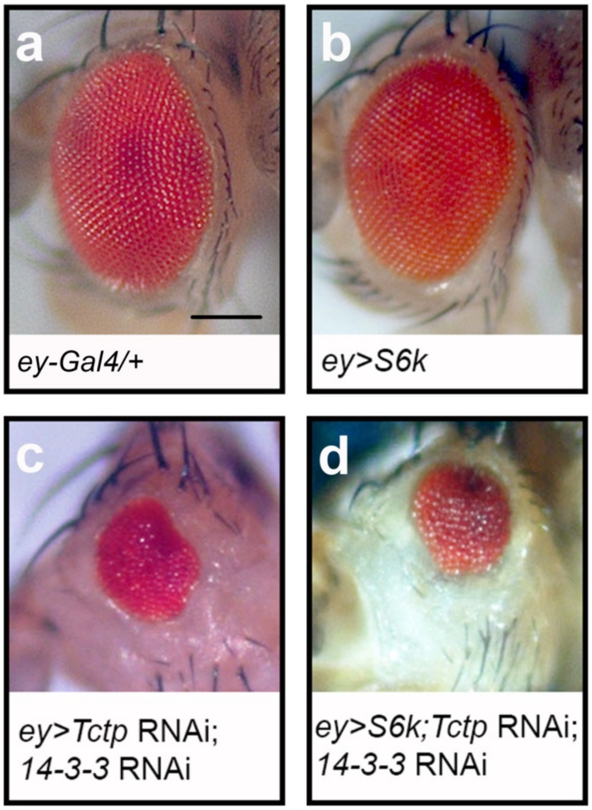

Supplementary Figure 4. Overexpression of S6k cannot rescue the phenotype of double knockdown of Tctp and 14-3-3. a, Fly with one copy of *ey-Gal4* has normal eye. **b,** Overexpression of S6k by *ey-Gal4* has no effect on eye size. **c,** Double knockdown of Tctp and one isoform of 14-3-3 results in strong reduction of eye size. **d,** Overexpression of S6k cannot rescue the small eye phenotype caused by knockdown of both Tctp and 14-3-3ε or 14-3-3ζ. Scale bar, 200 μm (**a-d**).

Supplementary Figure 5

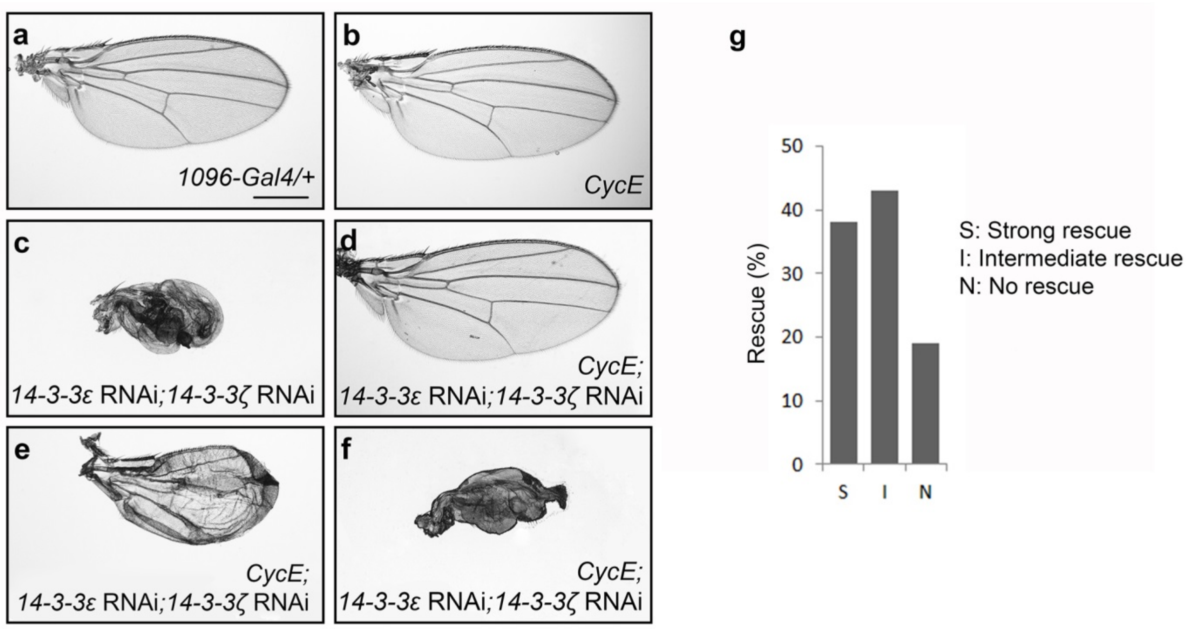

Supplementary Figure 5. CycE suppresses wing defects caused by double knockdown of both 14-3-3 forms. Effects of CycE overexpression on 14-3-3s depleted wings. *MS1090-Gal4/+* control (**a**) and CycE overexpression (**b**) show normal wings. **c**, Double knockdown of both 14-3-3 isoforms results in severe loss of wing tissues (<20% of wild-type wing size). **d-f,** Overexpression of CycE suppresses the 14-3-3 double knockdown phenotype, showing strong rescue (61-100% of wild type size) (**d**), intermediate rescue (21-60%) (**e**), or no rescue (20% or below) (**f**). (**g**) Quantification of the data shown in (**d-f**). 60 flies with the genotype of *MS1096>14-3-3ε* RNAi/+; *14-3-3ζ* RNAi/+; *CycE/+* were scored. Scale bar, 400 μm (**a-f**).

Supplementary Figure 6

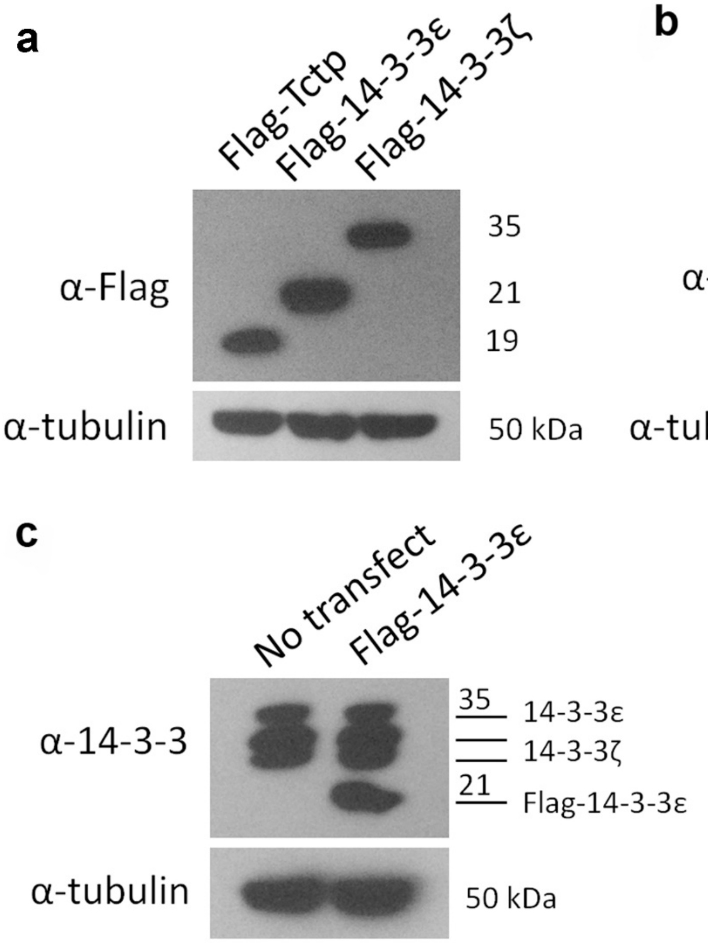

Supplementary Figure 6: Stoichiometric ratio between the levels of endogenous 14-3-3s and overexpressed Tctp and Rheb. a-b, Relative levels of transfected Flag-14-3-3ε, Flag-14-3-3ζ and Flag-Tctp (**a**) or Flag-Rheb (**b**) were examined with anti-Flag antibody. Three proteins show similar expression levels. **c,** Endogenous 14-3-3s in non-transfected cells and Flag-14-3-3ε in transfected cells show comparable levels, as tested by anti-14-3-3 antibody. These data suggest

that levels of endogenous 14-3-3s are not significantly different from the overexpressed Tctp/Rheb.

Supplementary Figure 7

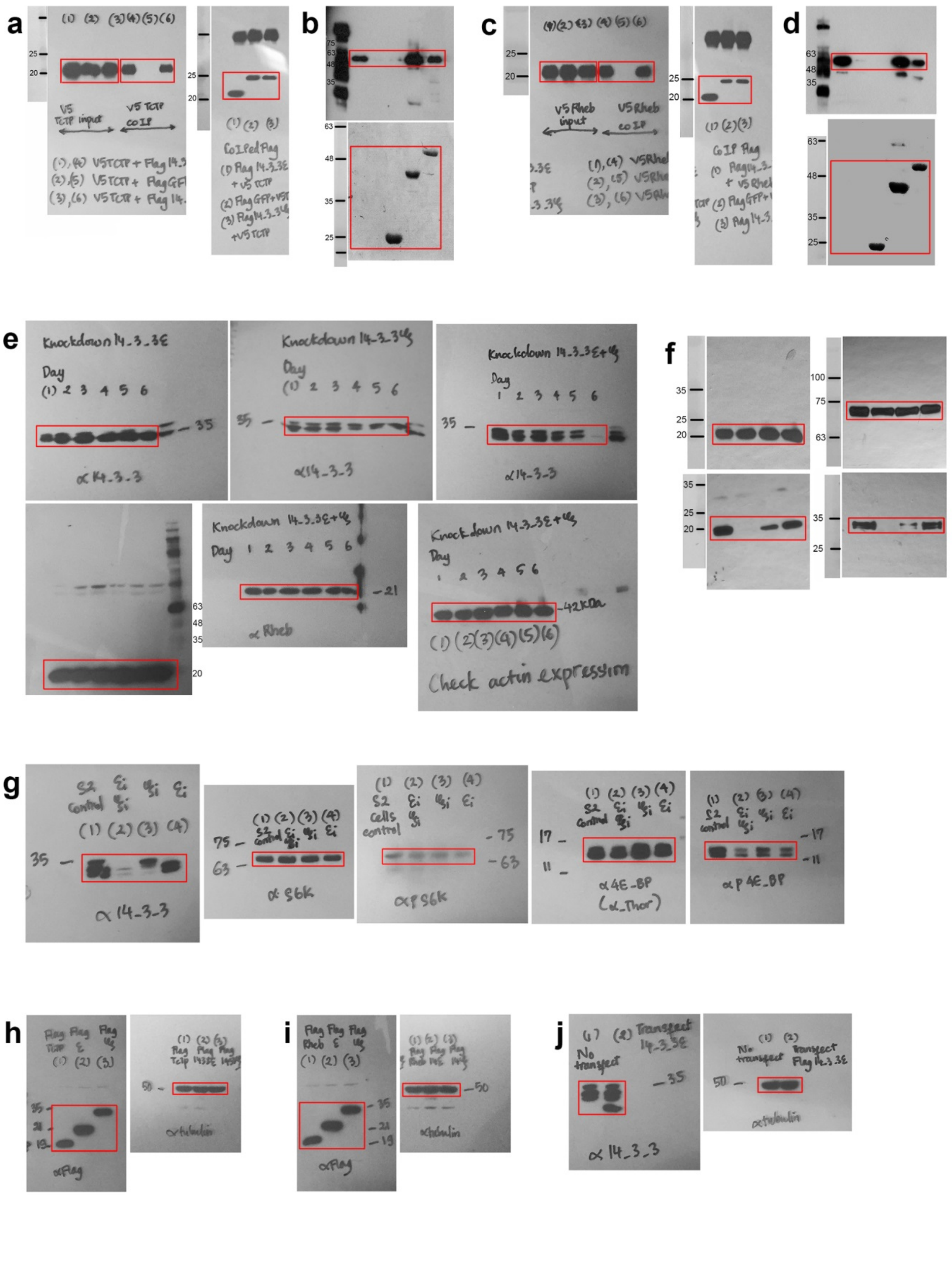

Supplementary Figure 7. Uncropped Western blots. a, Uncropped blots shown in Figure 4a. **b,** Blots shown in Figure 4b. **c,** Blots shown in Figure 4c. **d,** Blots shown in Figure 4d. **e,** Blots shown in Figure 5a. **f,** Blots shown in Figure 5b. **g,** Blots shown in Figure 6a. **h,** Blots shown in Supplementary Figure 6a. **i,** Blots shown in Supplementary Figure 6b. **j,** Blots shown in Supplementary Figure 6c.
